# Identification of CXCL5 expression as a predictive biomarker associated with response and prognosis of immunotherapy in patients with non‐small cell lung cancer

**DOI:** 10.1002/cam4.4567

**Published:** 2022-02-12

**Authors:** Jie Deng, Xuejiao Ma, Yang Ni, Xiaomin Li, Wenjing Xi, Minqi Tian, Xing Zhang, Manyu Xiang, Wanglong Deng, Chao Song, Hao Wu

**Affiliations:** ^1^ Department of Oncology The First Affiliated Hospital of Nanjing Medical University Nanjing Jiangsu China; ^2^ State Key Laboratory of Translational Medicine and Innovative Drug Development Jiangsu Simcere Diagnostics Co., Ltd. Nanjing Jiangsu China; ^3^ Department of Medicine Nanjing Simcere Medical Laboratory Science Co., Ltd. Nanjing Jiangsu China

**Keywords:** biomarker, CXCL5, immunotherapy, non‐small cell lung cancer, tumor microenvironment

## Abstract

**Background:**

The breakthrough of immunotherapy has revolutionized the treatment of non‐small cell lung cancer (NSCLC). However, only a limited part of patients could derive clinical benefits. To study how immune microenvironment (IME) of patients could influence the therapeutic efficacy of immunotherapy, we evaluated the response patterns of NSCLC patients treated with PD‐1 inhibitors and analyzed the molecules related to prognosis and efficacy of immunotherapy.

**Methods:**

Tumor samples were collected from 47 NSCLC patients treated with PD‐1 inhibitors. RNA expressions of tumor immune‐related 289 genes were analyzed using NanoString nCounter. Immune infiltration and correlation between clinical information and expression of immune‐related genes were assessed.

**Results:**

Unsupervised clustering analysis revealed two groups infiltrated with different immune cells and differentially expressed genes (DEGs) including CXCL5, CXCL9, IDO1, and LAG3 were found between groups. Stratification based on DEGs indicated that the group with high expression of CXCL5 was characterized by neutrophils. Univariate and multivariate Cox analysis further demonstrated that CXCL5 mRNA expression was positively associated with worse progression free survival (PFS). Logistic analyses indicated high CXCL5 was associated with worse response to immunotherapy.

**Conclusions:**

CXCL5 may be a potential biomarker for prognosis and responsiveness to immunotherapy and may be a novel preventive and therapeutic target for NSCLC.

## Graphical abstract

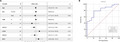

## 1 INTRODUCTION

Lung cancer is the second most frequent cancer and remains the leading cause of cancer‐related death, with approximate 2.2 million new cases and 1.8 million deaths worldwide in 2020 and about 85% cases are diagnosed with non‐small cell lung cancer (NSCLC),^1^, ^2^ which usually does not show obvious clinical symptoms until in the late stage. Delayed diagnosis with advanced disease leads to a low 5‐year survival rate of less than 15%.^3^

Since nivolumab and pembrolizumab as the blockade of programmed cell death‐1 (PD‐1) were approved by US Food and Drug Administration in 2015, immune checkpoints inhibitors (ICIs) have brought a new era of immunotherapy in NSCLC.^4^ In several clinical trials ICIs demonstrated improvement in overall survival (OS) in a limited number of patients either as monotherapy or combined with chemotherapy.^5^, ^6^, ^7^ When taken programmed death ligand 1 (PD‐L1) expression on more than 50% tumor cells as preselection, the objective response rate to immunotherapy was still lower than 50% in NSCLC.^8^ And “low” PD‐L1 expression (<1% tumor proportion score) could not totally exclude the patients who benefited from ICIs. Various predictive biomarkers have been explored like tumor mutational burden (TMB),^9^ driver gene mutations,^10^ but none have been enough validated to discriminate the patients sensitive to ICIs in clinical application.

Tumor microenvironment (TME) is a promising area of research with studies suggesting immune gene signatures as biomarkers for prognosis and response to ICIs. High levels of tumor infiltration lymphocytes (TILs) containing CD3^+^, CD4^+^, and CD8^+^ are associated with prolonged survival in NSCLC.^11^ Some studies concentrating on interferon gamma (IFNγ) relate to mRNA signatures, which are involved in many processes of immune responses and predictive for a higher efficacy of ICIs.^12^, ^13^ Some gene expression signatures related to immune genes also show their potential utility as biomarkers in immunotherapy.^14^, ^15^, ^16^

In an attempt to understand the complex IME in NSCLC patients treated with ICIs and discover more explicit predictive biomarkers, we evaluated the response patterns of these patients and analyzed the molecules related to prognosis and efficacy of immunotherapy.

## 2 MATERIALS AND METHODS

### 2.1 Patients

This study included the patients treated with anti‐PD‐1 as monotherapy or combined with chemotherapy from The First Affiliated Hospital of Nanjing Medical University between October 2018 and February 2020. Written informed consent was obtained from all patients before enrollment and study protocol was approved by the Institutional Review Board of The First Affiliated Hospital of Nanjing Medical University (No. 2021‐NT‐13). Clinicopathologic features and treatment histories of patients were extracted from medical records. Samples with failed quality control (QC) or patients evaluated as stable disease for less than 24 weeks follow‐up were excluded.

### 2.2 RNA sequencing and data processing

All tumor samples were acquired before anti‐PD‐1 treatment and preserved formalin‐fixed paraffin‐embedded slides. Gene expression was measured using the nCounter platform (NanoString Technologies) and transcriptome analysis was based on the 289‐immuno‐gene panel. This panel allows simultaneous analysis of 289 genes involved in the immune response in cancer. For each sample, QC indicators included the imaging QC, binding density QC, positive control linearity QC and positive control limit of detection QC. Positive normalization factor and content normalization factor were then calculated. Samples qualified for QC were included in subsequent analysis. The raw data of each sample and genes were standardized against internal controls to eliminate technical variability in the assay, and then counts were normalized to the geometric mean of endogenous housekeeping genes followed by log2 transformation.

### 2.3 Estimation of TME cell infiltration

Marker genes of 14 immune cell types, including B‐cells, dendritic cells, macrophages, exhausted CD8 T cells, CD8 T cells, neutrophils, mast cells, cytotoxic cells, Treg, natural killer CD56dim cells, NK cells, CD45, and Th1 cells were retrieved from the method previously reported.^15^, ^17^, ^18^ We further divided the macrophages into M1 and M2 macrophages according to the previous reports.^19^, ^20^ The role of M1 macrophages is to secrete pro‐inflammatory cytokines and chemokines, present antigens, participate in a positive immune response and act as an immune monitor. M2 macrophages can reduce inflammation, promote tumor growth, and immunosuppress. All TME cell infiltration scores were calculated as the arithmetic mean of constituent genes.

### 2.4 Generation of TME signatures

We constructed a set of gene sets that stored genes associated with some biological processes, including cytotoxic T lymphocyte (CTL) levels, cytolytic activity (CYT) score, chemokines, T cell markers, total TIL score, Teff score, IFN‐γ signature, and GEP score. The CYT score of each sample was evaluated based on the geometric mean of the product of *PRF1* and *GZMA* genes.^14^ GEP score was calculated as a weighted linear average of the constituent genes,,^13^, ^21^ and the remaining TME signatures were calculated as the arithmetic mean of corresponding genes.^12^, ^15^, ^16^, ^22^, ^23^

### 2.5 Unsupervised consensus clustering

In this study, we used nonnegative matrix factorization of R package to virtually dissect the RNA expression profiles of 47 patients and extract the immune–related expression pattern to characterize the immune landscape of NSCLC.^24^ The value of k where the magnitude of the cophenetic correlation coefficient began to fall was chosen as the optimal number of clusters (Figure S1). We classified the patients into two groups based on the immune expression, namely the TMECluster1 and TMECluster2. The number of clusters and their stability were determined by the consensus clustering algorithm. We used the ConsensuClusterPlus package to perform the above steps and 200 times of repetitions were conducted for guaranteeing the stability of classification.^25^

### 2.6 Identification of DEGs between different clusters

To identify immune‐related genes, we classified the patients into two distinct TME Clusters, namely the DEGCluster1 and DEGCluster2. The empirical approach of limma and edgeR package was applied to determine DEGs between the two TME Clusters.^26^, ^27^ Specifically, according to the read count of the sample, the TMM standardization method of the calcNormFactors function in the edgeR package was used. After log‐transform, the limma‐trend method in the limma package was employed for differential analysis using the Benjamini–Hochberg method. The significant criteria for determining DEGs was set as *p* values <0.05 and expression fold change (FC) ≥2 or ≤1/2.

### 2.7 Evaluation of the efficacy

Time from the first day of immunotherapy to progression or the last time of follow‐up was considered as progression‐free survival (PFS). The clinical response was estimated in accordance with the response evaluation criteria in solid tumors.^28^ In our study, the patients with complete response, partial response, and stable disease for at least 24 weeks after use of anti‐PD‐1 agents were defined as responders, while those with progressive disease were as nonresponders.

### 2.8 Statistical analysis

The unpaired *t* test was used to compare the estimated immune cell types and immune signature scores between two groups. Survival analyses were performed with Kaplan–Meier curves and log‐rank test. Univariate and multivariate Cox proportional hazard regression models were constructed to adjust for confounding variables including age, gender, TNM‐based staging, and expression of DEGs. To test the correlation between responsiveness to anti‐PD‐1 agents and variables, a logistic regression model was used.

All statistical analyses were performed using R package (version 3.5.1) and SPSS software (version 22.0, Chicago, IL). Boxplots were generated in R with the ggplot2 package, indicate median and interquartile range. Statistical tests were two‐sided and with *p* value <0.05 taken as statistical significance.

## 3 RESULTS

### 3.1 Patient characteristics

After exclusion of three patients with unqualified samples and four SD patients with less than 24 weeks follow‐up time, a total of 47 eligible patients were analyzed in the study (Table 1). The median follow‐up was 11 months (range, 0 to 31 months). Patients were 43–81 years old and with a male predominance. Eleven of 47 patients (23%) were treated with anti‐PD‐1 agents as monotherapy and the rest (77%) received immunotherapy combined with chemotherapy. There were 12 patients with stage III, 33 with stage IV, and 2 with unknown stage. Based on TNM staging, the patients were divided into two groups except for those with unknown stage. The results showed no differences between these two groups in baseline characteristics (all *p* > 0.05; Table 1).

**TABLE 1:** Baseline characteristics of patients (*n* = 47)

Characteristics	All, *n* (%)	Stage III, *n* (%)	Stage IV, *n* (%)	*p* value
*N* ^a^	47 (100)	12 (26)	33 (70)	
Age, years				
Median (range)	67 (43–81)	66.5 (55–81)	67 (43–80)	0.738
Sex				
Female	8 (17)	1 (8)	6 (19)	0.655
Male	39 (83)	11 (92)	27 (81)	
Smoking				
Never smoker	16 (34)	3 (25)	12 (36)	0.051
Former smoker	24 (51)	4 (33)	19 (58)	
Current smoker	6 (13)	4 (33)	2 (6)	
NA	1 (2)	1 (8)	0	
Pathology				
LUAD	18 (38)	2 (17)	15 (45)	0.090
LUSC	28 (60)	10 (83)	17 (52)	
NA	1 (2)	0	1 (3)	
ECOG				
0	15 (32)	5 (42)	10 (30)	0.796
1	30 (64)	7 (58)	22 (67)	
2	1 (2)	0	1 (3)	
NA	1 (4)	0	0	
Immunotherapy				
Monotherapy	11 (23)	2 (17)	9 (27)	0.168
Combined with chemotherapy	36 (77)	10 (83)	24 (73)	
Drug				
Camrelizumab	13 (28)	3 (25)	8 (24)	0.565
Nivolumab	11 (23)	1 (8)	10 (30)	
Pembrolizumab	9 (19)	3 (25)	6 (19)	
Toripalimab	7 (15)	2 (17)	5 (15)	
Sintilimab	7 (15)	3 (25)	4 (12)	

Abbreviations: ECOG, Eastern Cooperative Oncology Group; LUAD, lung adenocarcinoma; LUSC, lung squamous cell carcinoma; NA, not available.

^a^ Two patients with unknown stage were excluded. *P* values were based on Fisher's exact test or Mann–Whitney test.

### 3.2 TME subtypes

**FIGURE 1:**
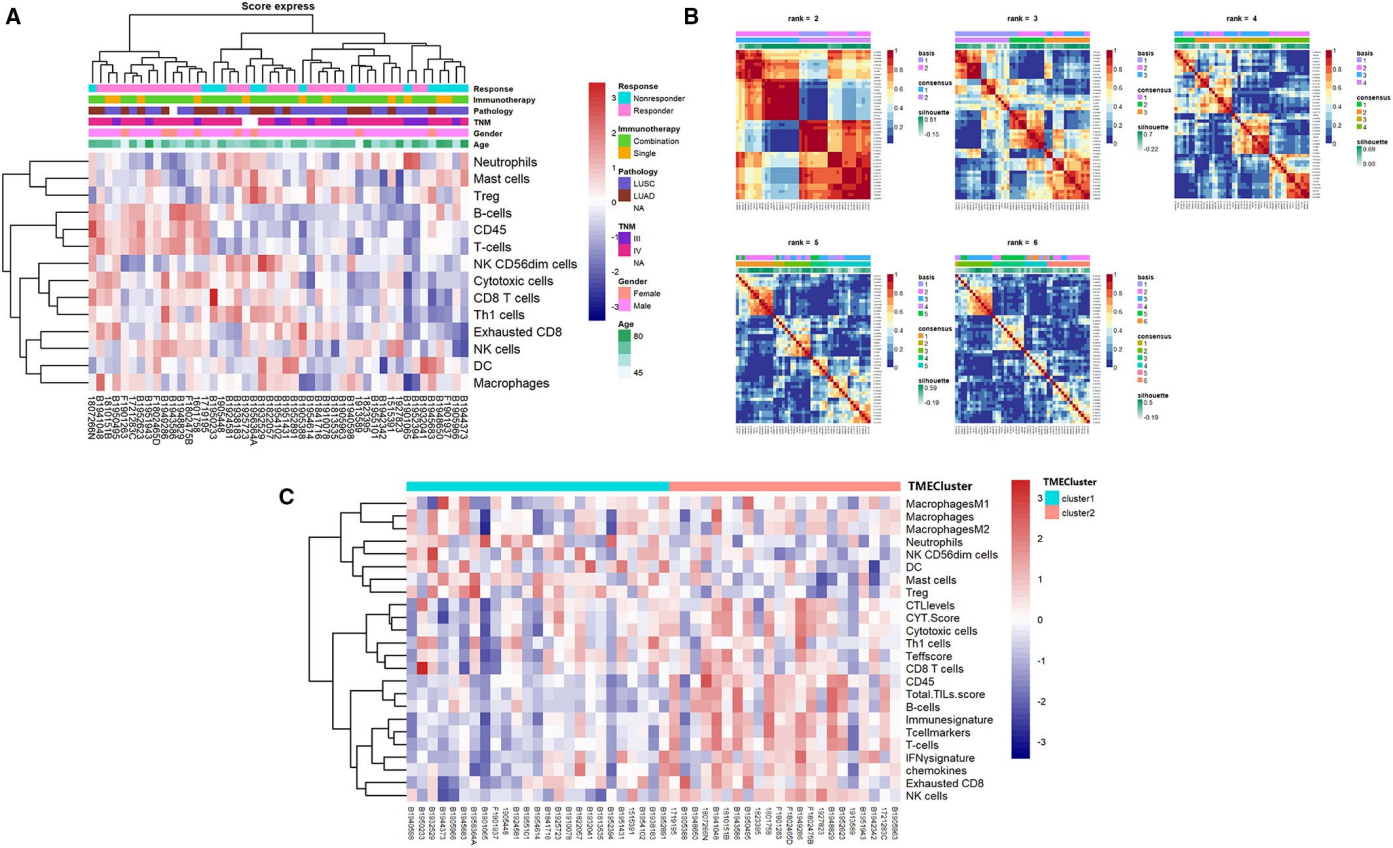
Unsupervised clustering based on TME and characteristics of TMEclusters. (A) Unsupervised clustering of 14 cells for patients. TNM stage, pathology, age, gender, immunotherapy, and response are annotated. (B) Cluster number of two is optimal. (C) Immune cell infiltration and signatures of the two TMEclusters

To discriminate different patterns of TME, we performed an unsupervised consensus clustering based on 14 immune infiltration cell types (Figure 1A). The results revealed the cluster number of two was optimal (TMECluster1: *n* = 25; TMECluster2: *n* = 22) and microenvironment cell infiltration and immune signatures of the two clusters were demonstrated in Figure 1B. TMECluster1 was poor in TILs like B cells, T cells, and NK cells, with low levels of immune signatures, whereas TMEcluster2 was characterized by high infiltration in cytotoxic cells, chemokines, and high CYT scores and total TIL scores Figure 1C. Through differential expression analysis between the two clusters, four DEGs including CXCL5, IDO1, CXCL9, and LAG3 were acquired.

### 3.3 DEG‐based subtypes

**FIGURE 2:**
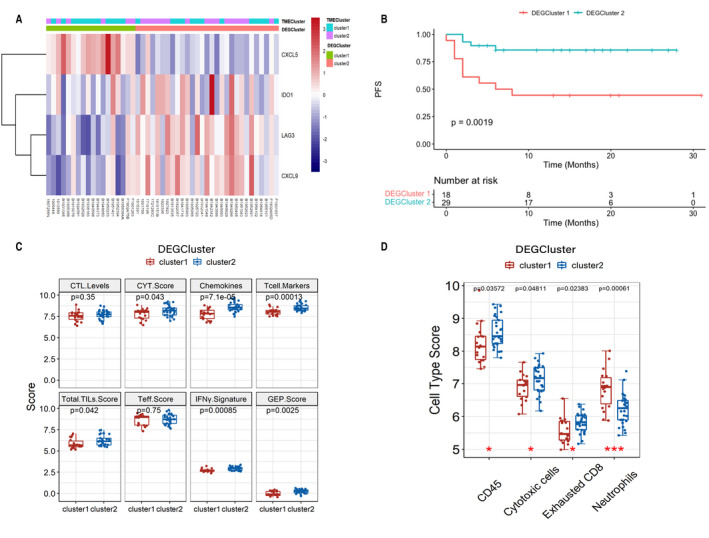
Construction of DEGClusters with unsupervised clustering based on DEGs and characteristics of DEGClusters. (A) Unsupervised analysis and clustering of DEGs. (B) Kaplan–Meier curves for two DEGClusters. (C, D) immune scores and components of infiltrated immune cells of two DEGClusters

Unsupervised clustering analysis of expression of four DEGs was utilized to stratify patients into two clusters, DEGCluster 1 (*n* = 18) and DEGCluster 2 (*n* = 29). The matching rate of the TMEClusters and DEGClusters was 48.0% and 72.7% for DEGCluster1 and DEGCluster2 (Figure 2A).

We further investigated the differences in the immune microenvironment between two clusters. The immune and cell type scores were measured to quantify the infiltration of immune cells in tumors. DEGCluster2 was characterized by relatively high immune scores related to cytolytic activity, chemokines, T cells, TILs, and IFNγ signatures (Figure 2C). Higher abundance of CD45 cells, cytotoxic cells, and exhausted CD8 cells, but lower levels of neutrophils were observed in DEGCluster2 (Figure 2D). And Cluster2 exhibited superior survival (Figure 2B).

### 3.4 Association between expression of DEGs and PFS

**FIGURE 3:**
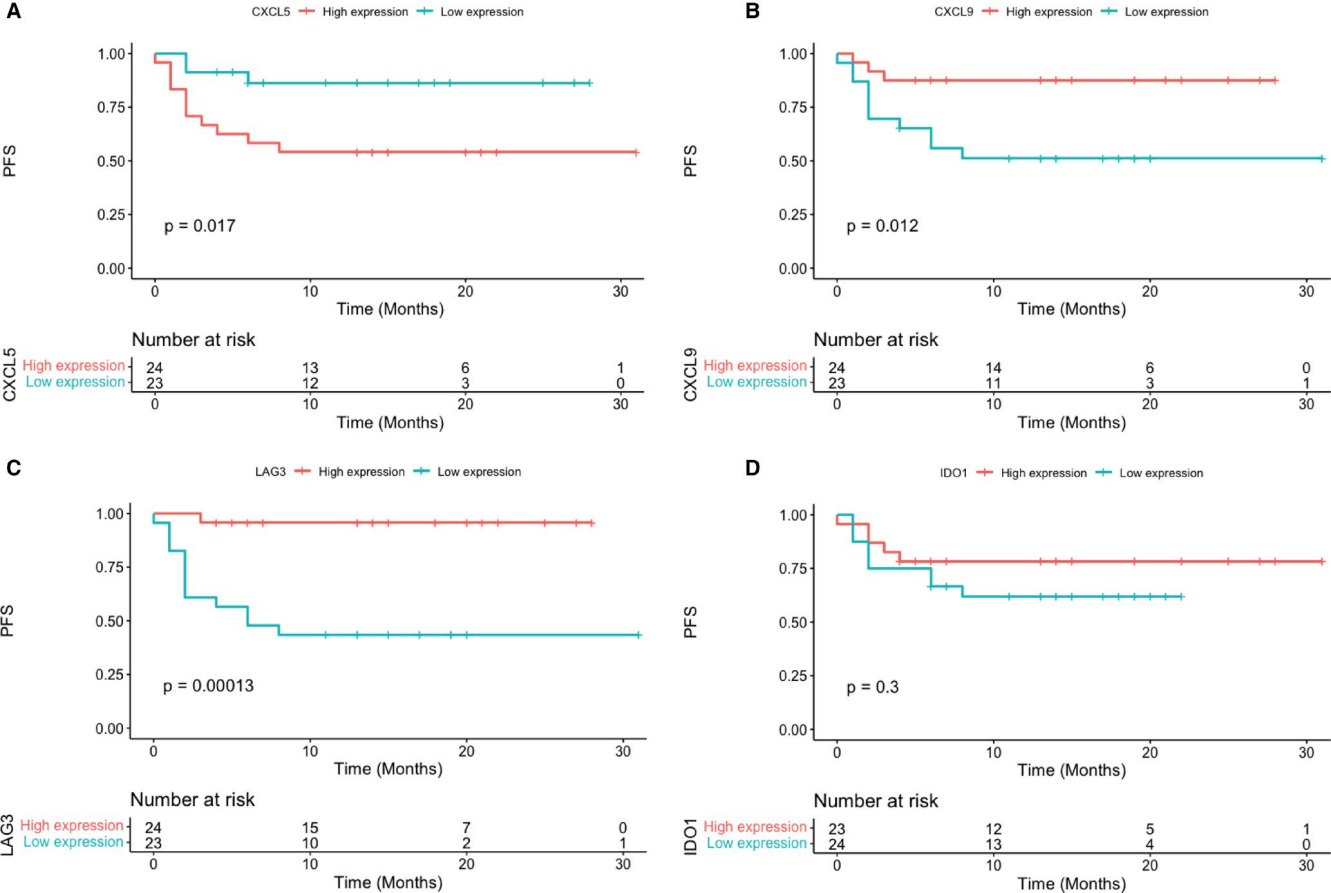
Kaplan–Meier survival analysis for high‐ and low expression of (A) CXCL5, (B) CXCL9, (C) LAG3, and (D) IDO1

Using the Kaplan–Meier method, a statistically significant longer PFS was shown for patients with lower CXCL5 expression (*p* = 0.017) (Figure 3A). On the contrary, patients with higher expression of CXCL9, LAG3 had a prolonged PFS (*p* = 0.012 and <0.001, respectively) (Figure 3B,C). However, no significant correlation was observed between expression of IDO1 and PFS (*p* = 0.3) (Figure 3D).

To further investigate the association between PFS and other clinicopathological variables, univariate and multivariate Cox proportional hazards models were constructed. The univariate analysis showed the expression of CXCL5 (*p* < 0.001), IDO1 (*p* = 0.022), CXCL9 (*p* = 0.019), and LAG3 (*p* = 0.002) was statistically associated with survival. After adjusting for clinical factors including age, sex, and TNM stage, multivariable analyses revealed that expression of CXCL5 (hazard ratio: 1.791, 95%CI: 1.232–2.603; *p* = 0.003) was an independent prognostic factor for PFS (Table 2).

**TABLE 2:** Univariate and multivariate Cox analysis of the expression of DEGs and clinicopathological characteristics

Variables	Univariate analysis			Multivariate analysis		
	HR	95%CI	*p* value	HR	95%CI	*p* value
Age	1.035	0.971–1.103	0.295	1.022	0.941–1.108	0.607
Gender						
Male vs. female	1.401	0.313–6.268	0.659	1.838	0.112–30.206	0.670
TNM						
IV vs. III	2.213	0.490–9.995	0.302	3.178	0.526–19.193	0.208
CXCL5	1.829	1.353–2.473	<0.001*	1.791	1.232–2.603	0.003*
IDO1	0.532	0.310–0.915	0.022*	0.535	0.279–1.027	0.060
CXCL9	0.635	0.435–0.927	0.019*	0.873	0.408–1.867	0.726
LAG3	0.405	0.230–0.715	0.002*	0.823	0.359–1.887	0.646

Abbreviations: HR, hazard ratio; CI, confidence interval.

* *p* < 0.05.

### 3.5 Predictive signatures in response

**FIGURE 4:**
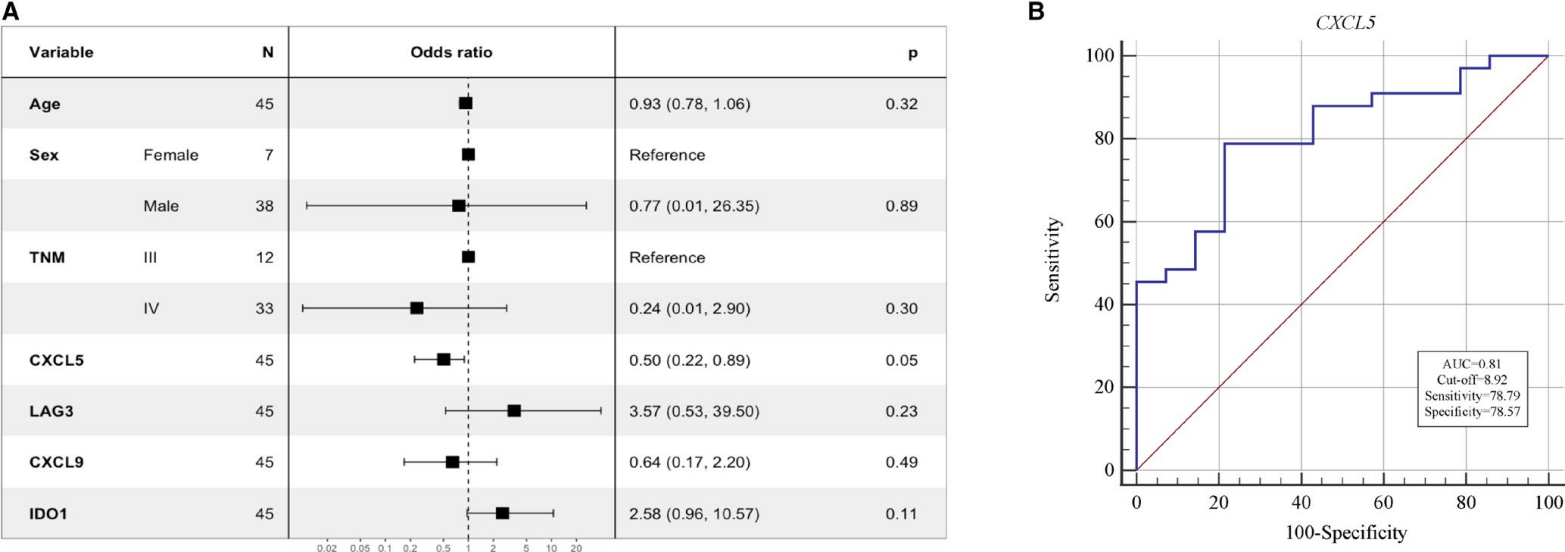
Association between CXCL5 expression and response to immunotherapy. (A) Logistic regression of DEG expression and clinicopathologic characteristics. (B) Receiver operating characteristic curve for the prediction of response to immunotherapy

After identification of prognostic value of CXCL5 expression, we conducted Logistic regression analyses to further evaluate different variables in predicting response. According to assessment to anti‐PD‐1 treatment, the response rate was 70.2%. There was a correlation between response to ICI treatment and expression of CXCL5 (Odds radio: 0.5, 95%CI: 0.22–0.89, *p* = 0.05) (Figure 4A).

The receiver operating characteristic curve was drawn based on whether the expression of CXCL5 was used as a predictor for short‐term benefit (dichotomy of response and nonresponse). The area under the curve was 0.81 with the optimal cutoff value of 8.92. The specificity was 78.57% and the sensitivity was 78.79%, implying a moderately high ability of the CXCL5 expression to predict the response to immunotherapy.

## 4 DISCUSSION

In this analysis, we performed a comprehensive analysis of TME to identify immune‐related biomarkers to predict the responsiveness to immunotherapy and prognosis of NSCLC patients. Based on immune scores and DEGs we divided the patients into two clusters and compared the difference of immune components between them. The results found that an upregulation of expression of CXCL5 was an independent predictor for poor prognosis and unfavorable response to ICIs.

Innate and adaptive immune cells play an essential role in TME remodeling and could be predictors for clinical outcomes.^29^ The effect of TILs on prognosis is controversial since they may participate in tumor‐promoting or tumor‐suppressing activities which lead in the different stages of tumor progression. High levels of TILs are mainly correlated with better outcomes.^30^ CD8^+^ cytotoxic T cells evoke the tumor destroying by releasing IFNγ and other factors, and there is a positive correlation between high infiltration of CD8+ lymphocytes and prolonged survival in NSCLC.^31^, ^32^ Consistent with our results an increased inflammatory microenvironment has been shown higher level of cytotoxic cells and higher score of IFNγ signature in DEGCluster2, which demonstrated favorable PFS. And enriched exhausted CD8+ cells in the same group could be explained by persistent antigenic stimulation.^33^

CXCL5, a pro‐inflammatory cytokine, belonged to the CXC‐type chemokine family, which was secreted by tumor or tumor infiltrating immune cells and regulated the TME.^34^, ^35^ Combined with chemokine receptor 2 (CXCR2), CXCL5 participated in recruiting leukocytes, proliferating tumor cells, and metastasis. CXCL5 promotes cell proliferation and migration by activating the downstream MAPK/ERK1/2 and PI3K/AKT signaling pathways, in which activation of AKT and ERK1/2 has influence on the elevated activity of neutrophils in NSCLC.^36^ Tumors with high expression of CXCL5 were highly infiltrated with T cells and macrophages, which took part in immune responses.^37^ Besides NSCLC, several previous studies showed that increased expression level of CXCL5 was an adverse prognosis biomarker in pancreatic cancer, hepatocellular carcinoma, and bladder cancer.^38^, ^39^, ^40^ Congruously, we found that the high expression of CXCL5 group was enriched in neutrophils and demonstrated shorter PFS. In addition, a negative correlation between CXCL5 expression and response to anti‐PD‐1 agents was proved.

To the best of our knowledge, we first reported the CXCL5 expression level as an independent factor for responsiveness to ICIs. The results were validated in another cohorts with lung cancer treated with immunotherapy.^41^ A higher responder rate was observed in the patients with low CXCL5 expression levels compared with high expression levels, but the difference was statistically insignificant due to the limited sample size, suggesting that CXCL5 expression might be a potential predictive and prognostic biomarker for immunotherapy in NSCLC. Currently, CXCL5 has also been proved to contribute to angiogenesis and proliferation, promoting malignant progression in distinct cancers.^37^ CXCL5 expression was positively associated with the risk of metastasis in bladder cancer, NSCLC, and melanoma.^40^, ^42^, ^43^ Inhibitors of CXCL5 or its receptor CXCR2 could be potentially applied in clinic in different mechanisms, such as regulating the tumor immune microenvironment, reducing angiogenesis and progression, improving treatment utility by combination therapy.^37^

There were several limitations in our study that should be concerned. First, this was a retrospective analysis of a relatively small cohort of pre‐treated patients who received anti‐PD‐1 treatment in a real world. Instead of clinical trials, the confounding effects of prior therapies of patients were uncontrollable and incomparable. Second, the research was based on transcriptomic data, further multi‐omics data integration or functional experiments are needed to verify the mechanisms of CXCL5.

In conclusion, our study found a novel prognosis‐related gene clustering based on four genes, CXCL5, LAG3, IDO1, and CXCL9. This clustering was associated with tumor immune infiltration. Furthermore, increased CXCL5 expression was related to unfavorable survival and poor responsiveness to ICIs. Therefore, CXCL5 is likely to exert a crucial impact on TME and has the potential to act as a prognostic biomarker for prognosis and response to immunotherapy in NSCLC.

## CONFLICT OF INTEREST

The authors declare that they have no competing interest.

## AUTHOR CONTRIBUTIONS

J. Deng and H. Wu were responsible for study conception. J. Deng, X. Ma, and Y. Ni wrote the original draft. X. Li, W. Xi, W. Deng, and X. Zhang analyzed and interpreted the data. M. Tian, M. Xiang, and C. Song participated in sample collection and investigation. H. Wu reviewed and edited the manuscript. All the authors read and approved the final manuscript.

## CONSENT FOR PUBLICATION

Written informed consent was obtained from all patients before enrollment.

## ETHICS APPROVAL AND CONSENT TO PARTICIPATE

The study protocol was approved by the Institutional Review Board of The First Affiliated Hospital of Nanjing Medical University (No. 2021‐NT‐13).

## Supporting information


Figure S1

## Data Availability

The datasets used and/or analyzed during the current study are available from the corresponding author upon reasonable request.
