# Supplementary figures and images for: Identification of CXCL5 expression as a predictive biomarker associated with response and prognosis of immunotherapy in patients with non‐small cell lung cancer
Source: Cancer Med. 2022 Feb 12;11(8):1787–95. doi: 10.1002/cam4.4567 (PMC9041069; doi:10.1002/cam4.4567)

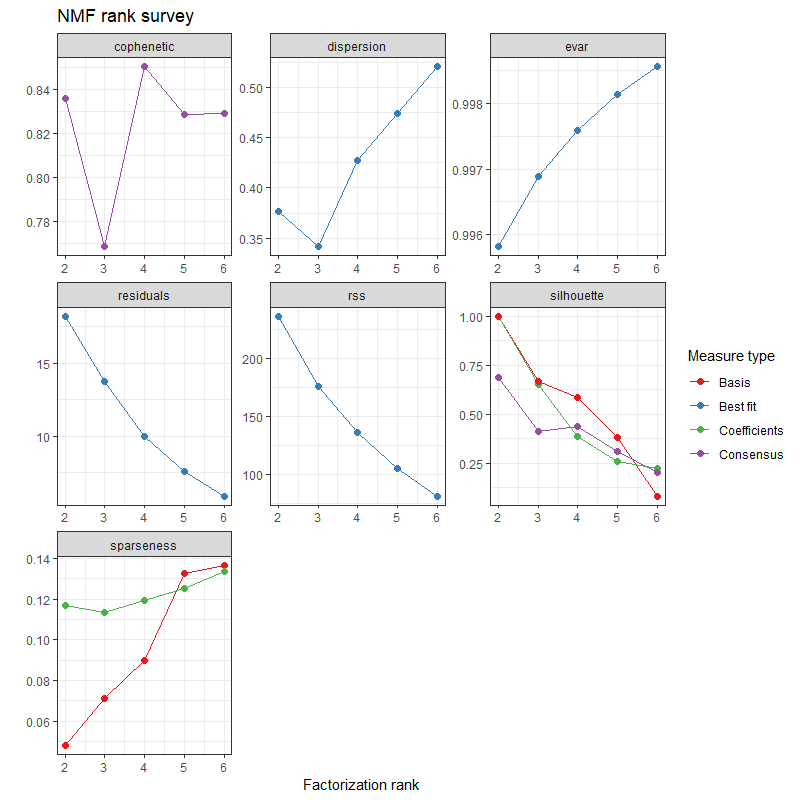

Supplement: Supplementary file 1 — Figure S1 [file CAM4-11-1787-s001.tif]
